# Application of Photodynamic Therapy in Pediatric Dentistry: Literature Review

**DOI:** 10.3390/pharmaceutics15092335

**Published:** 2023-09-18

**Authors:** Tamiris Silva, Ana Júlia Lacerda Lunardi, Ana Carolina Santos Menezes Barros, Amanda Rafaelly Honório Mandetta, Elizabeth Grudzien, Magdalena San-Martín, Anna Carolina Ratto Tempestine Horliana, Sandra Kalil Bussadori, Lara Jansiski Motta

**Affiliations:** 1 Postgraduate Program in Biophotonics Applied to the Health Sciences, Nove de Julho University, São Paulo 01525-000, SP, Brazilsandra.skb@gmail.com (S.K.B.); 2 Departamento de Bienestar y Salud, Universidad Católica del Uruguay, Av. 8 de Octubre 2738, Montevideo 11600, Uruguay

**Keywords:** photodynamic therapy, pediatric dentistry, oral health, dental care

## Abstract

Microbiological control of dental pathologies presents a significant clinical challenge for dental surgeons, particularly considering drug-resistant microorganisms. To address this issue, Antimicrobial Photodynamic Therapy (PDT) has emerged as an effective and complementary technique for microbial reduction. This therapy involves the application of a photosensitizer dye (PS) either topically or systemically, followed by exposure to low-power lasers with appropriate visible light wavelengths. PDT has found a valuable place in dentistry across various specialties, including surgery, periodontics, endodontics, dentistry, implantology, orthodontics, and pediatrics. In the realm of pediatric dentistry, managing microorganisms during dental treatments has become a major challenge. Considering its promising results and ease of application, Photodynamic Therapy presents an interesting alternative for clinical practice. However, it is important to note that specific protocols must be followed for each application, encompassing the type of photosensitizer, concentration, pre-irradiation time, light type, wavelength, energy, power, and mode of light delivery. Researchers have been steadily refining these protocols to facilitate PDT’s integration into clinical practice. The objective of this review is to describe in which procedures and oral health problems in children PDT can be applied. In this sense, we list what the literature brings about the possibilities of applying PDT in a pediatric dentistry clinic.

## 1. Introduction

Antimicrobial Photodynamic Therapy (PDT) is a promising clinical treatment modality with diverse applications in eliminating unwanted cells and tissues, including the inactivation of microorganisms such as viruses, bacteria, and fungi. Its effectiveness in wound healing and tissue repair has shown positive outcomes in dental treatments [1,2,3]. PDT involves the combination of a photosensitizing agent (PS) and light in the presence of oxygen, leading to cytotoxic action [3]. The early increase in vascularization induced by light contributes to its beneficial effects [4]. Moreover, the primitive molecular nature of oxygen makes it less likely for microorganisms to develop resistance against this therapeutic approach [5].

PDT emerges as a novel alternative for controlling tissue proliferation, inflammation, and infection, particularly in easily accessible sites such as the skin and mucous membranes, which are common locations of pathogenic microorganism infections [6,7]. Furthermore, considering the pattern of infectious diseases and the rise of antibiotic-resistant bacterial strains, exploring new treatment approaches has become imperative [8,9].

Historically, Photodynamic Therapy (PDT) has its origins in the observation of endogenous porphyrin fluorescence during surgery, and the use of photosensitizing agents in microbial eradication dates to a time preceding the advent of chemotherapy [10,11]. The concept of combining a photosensitizing agent with light for treating tumors and pre-malignant diseases was pioneered by Paul Ehrlich around the turn of the 19th century, following extensive experiments on the effects of aniline dyes on animal and microbial cells [12,13].

PDT has found widespread acceptance in dentistry across various specialties, including surgery, periodontics, endodontics, dentistry, implantology, orthodontics, and pediatrics. Its applications are justified by its ability to promote rapid and effective healing, generate greater tissue repair in a short period, and reduce microbial load [14,15,16]. Additionally, PDT offers an accessible alternative that helps avoid overreliance on antibiotics in dentistry [15,17], thereby addressing the issue of bacterial resistance to these medications. [12,16].

At the core of PDT lies the principle that the interaction between light of the appropriate wavelength and a non-toxic photosensitizing compound, in the presence of oxygen, produces reactive species capable of inducing cell infeasibility [17,18,19]. Among the notable photosensitizing agents used in PDT, methylene blue stands out for its ability to absorb visible light [20,21,22]. These compounds play a crucial role in inducing or participating in photochemical reactions, rendering them useful as therapeutic agents [23,24,25].

**Figure 1:**
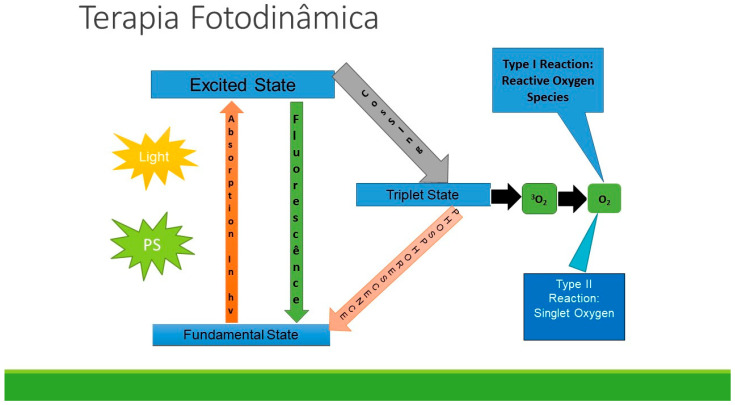
Jablonski Diagram.

The application of the photosensitizing agent occurs in the target tissue region that requires elimination, and the cytotoxicity is triggered only when the controlled beam of light incident upon these specific tissues, leading to the production of radicals through interaction with the photosensitizer [11,19,21]. The interaction between light and photosensitizers in PDT follows the mechanism proposed by Jablonski [26] Figure 1.

The initial excited singlet state of the PS undergoes an intersystem crossover to form the long-lived triplet state PS. This triplet state can pass through two parallel paths. Type I photochemistry, involving electron transfer reactions, can produce hydroxyl radicals. Type II photochemistry, involving energy transfer, can produce singlet oxygen. Both reactive oxygen species are cytotoxic for bacterial cells [21,22].

The initial excited singlet state of the PS undergoes an intersystem crossover to form the excited triplet state of PS, acting as a reactive intermediate to generate reactive oxygen species (ROS). When the PDT approach is used as an anti-infective or antimicrobial, it is generally referred to as photodynamic inactivation (PDI) [23]. ROS initiate additional oxidative reactions locally with the components of the bacterial cell wall, cell membranes, enzymes, or nucleic acids [24,25].

In view of the above, it is of great scientific interest to study PDT for the treatment of bacterial, viral, or fungal lesions and to review the literature in order to access adequate protocols for PDT as a potentially effective therapy for treatment in a localized way, with no systemic effects, and the possibility of it to exhibit the same action even in lineages of drug-resistant microorganisms [25,26].

To achieve successful Photodynamic Therapy (PDT), an appropriate and effective photosensitizer (PS) is crucial, which must possess specific characteristics, including a high resonant absorption coefficient with the wavelength of the light source, biological stability, photochemical efficiency, target cell selectivity, and minimal toxic effects on normal cells [26,27]. A photosensitizing substance acts as an optical absorption agent. Upon irradiation, the dye molecules enter an excited state, and the energy transferred from the light source to the dye results in the formation of reactive molecules, such as singlet oxygen, which can damage and eliminate bacterial cells [28].

Selecting the appropriate PS and light sources involves considering the PS’s behavior with light and its interaction with the target cells [21]. The PDT’s impact on cellular pathways depends on the PS’s location within the cell, influenced by its chemical nature, molecular weight, lipophilicity, amphiphilicity, ionic charge, protein-binding characteristics, PS concentration, incubation time, serum concentration, and the phenotype of the target cells [26,29]. It is important to note that the PS substance alone does not exhibit significant antimicrobial effects. However, upon irradiation with the appropriate visible light wavelength, the previously stable substance becomes unstable. During this process, photons (energy) are absorbed by the PS, leading to the generation of highly reactive and cytotoxic products in the presence of oxygen [7].

The energy transfer through the PS induces the spin inversion of one of the electrons in the oxygen molecule, converting it from a triplet to a singlet state. Singlet oxygen has a very short half-life (nanoseconds) and can only diffuse up to approximately 100 nm. Therefore, its cytotoxic activity is confined to the site of its production. To ensure successful photoactivation, a pre-irradiation period is necessary after the PS application so that the PS can accumulate in the target cell [30,31].

For PS to be considered an efficient photosensitizer, it must have the ability to absorb the light source, pass to an excited state and transfer this energy to molecular oxygen. Molecules presenting such characteristics are typically rigid planar structures with a high degree of conjugation [9,19,32]. The photosensitizers most used in PDT include phenothiazinium, porphyrin, and phthalocyanine, which have flat, simple, and tricyclic structures and are typically cationic in nature. The compounds most used in the medical field are methylene blue (MB) and toluidine blue (TBO); both are efficient in the production of singlet oxygen and present the maximum absorption of light with a wavelength of 656 nm for MB and 625 for TBO [27,33,34,35].

Phenothiazine-derived photosensitizers are the most used in PDT research. Phenothiazines are blue tricyclic heteroaromatic compounds, and the most used are toluidine blue and methylene blue. At low concentrations, they are sufficient to cause bacterial death and not damage cells such as keratinocytes and fibroblasts [36,37,38].

Phenothiazines are more effective against Gram-positive microorganisms compared to Gram-negative microorganisms. MB has been widely used for the treatment of endodontic microbiota microorganisms; it presents a light absorption band in a wavelength between 620 nm and 660 nm. Being of a hydrophilic nature, accompanied by a low molecular weight and positive charge, it prohibits a passage through porin protein channels in the outer membrane of Gram-negative bacteria. Furthermore, MB predominantly interacts with anionic lipopolysaccharide macromolecules, thus participating in the photosensitization process [32,39].

Since it was first synthesized in 1876, MB has been used in different areas of clinical medicine, such as chemotherapy and blood disinfection. Additionally, it also formed the basis of antimicrobial chemotherapy, particularly in the antimalarial area. Recently, the potential photosensitizing effect of MB and its congeners was recognized, leading them to be applied in association with light to produce an antimicrobial effect, especially in blood disinfection. The wide use of MB as a photosensitizing agent is related to the combination of its simple chemical structure with the ease of producing in situ oxidation–reduction reactions [12,40].

MB is of low toxicity, and it is a reduced side effects molecule, indicated by staining living organisms and treating methemoglobinemia, which is a disease characterized by an abnormal amount of methemoglobinemia in the blood [41]. Its main indications include the treatment of basal cell carcinoma, Kaposi’s sarcoma, melanoma, and viral and fungal infections. MB absorbs intense light in the therapeutic window and triggers mechanisms that damage biomolecules and induce death at several sites in target cells, tissues, and organisms [16,42].

In an in vitro study, the photosensitizer TBO showed a significant interaction with bacterial endotoxin (LPS) from Gram-negative bacteria. This interaction proved to be more effective compared to methylene blue PS, which could be one of the main factors that would explain the photooxidative effect against Gram-negative bacteria of toluidine blue [43].

Among the compounds that have been studied as photosensitizers in PDT for the treatment of cancer, in place of phenothiazines, is tannin [44,45,46]. Tannins are complex substances that derive from numerous vegetables and are classified as hydrolyzable and condensed [30]. Tannins are characterized by their astringency and their hemostatic effect. Therefore, their therapeutic applications are related to these properties. They are mainly used in the textile industry, as well as in the paint industry. They are used in laboratories for the detection of proteins and alkaloids and as antidotes in cases of alkaloid plant poisoning [47]. In order to measure the light absorption of hydrolyzable tannin, with the purpose of seeking new photosensitizers for use in PDT, the authors verified, through a spectroscopy study, that hydrolyzable tannin has an intense absorption band between 500 and 700 nm [48].

The hydrolyzable tannin has shown promising activity and no toxicity, and it has favorable photophysical properties, with high yields of the triplet state, in addition to proving to be a promising photosensitizer in tests using in vitro tumor cells, showing a strong absorption band at 672 nm and rapid skin elimination [49].

Topical anesthetics before irradiation should not be applied, as the acidic pH of anesthetics can chemically inactivate the photosensitizer. In general, PDT is well tolerated, and, in some cases, pain can be relieved by oral analgesics administered one hour before the procedure [50].

The objective of this review is to describe in which procedures and oral health problems in children PDT can be applied. In this sense, we list what the literature brings about the possibilities of applying PDT in the pediatric dentistry clinic.

## 2. Applications of PDT in Pediatric Dentistry

### 2.1. Caries

Early and severe childhood caries is a public health problem that requires great effort from health professionals who care for children [51]. Minimal intervention combined with knowledge about the development of dental caries has led to major transformations in the paradigm of restorative treatment, with a marked shift involving the maximum preservation of healthy dental tissue capable of remineralization [52].

Studies evaluating PDT in eliminating bacteria related to endodontic infections have increased in recent years. PDT has decontamination rates that reach 97–100%, matching those achieved with high-power lasers. The antimicrobial effect of PDT on endodontic pathogens (*P. gingivalis*, *P. intermedia*, *F. nucleatum*, *P. micros*, *P. endodontalis*) has been observed both in vitro and in vivo [32,53,54,55]. PDT has also been shown to be effective in reducing *E. faecalis* present in canals contaminated in vitro [32,56]. This is a very important result since this bacterium is related to persistent and refractory endodontic infections.

PDT has shown to be a promising alternative for antimicrobial treatment, and in this study, a technique was used to evaluate its effectiveness in the treatment of total viable bacteria, *Streptococci Mutans*, and *Lactobacillus* spp. Molar caries lesions were treated with 0.01% MB dye and irradiated with a low-power laser (660 nm; 100 mW; 320 Jcm^2^; 90 s; 9 J). Dentin samples from the pulpal wall region were collected using micropuncture before and immediately after treatment. The results found showed statistically significant reductions in Streptococcus Mutans, *Lactobacillus* spp., and total viable bacteria [51]. However, in another study, a decrease in the bacterial cultures tested was observed, but without statistically significant differences. The authors attributed the probability of this fact to the difference in dosimetry applied in comparison with other studies and the type of dentin substrate, which contributed to the differences between the findings of the study carried out with permanent teeth and this study, which used carious lesions in deciduous teeth [57].

*S. mutans* are bacteria present in the mouth and comprise 70% of the bacteria in dental plaque. Although dental caries is a multifactorial complication, *S. mutans* biofilms are the main cause of cavitated carious lesions. Considering the importance of this fact, Azizi et al. [58] evaluated several intervention possibilities using chlorhexidine (gold standard) and variations in the use of light and photosensitizers. After the intervention, the number of *Streptococcus* was reassessed, and, as a result, it proved to be an effective treatment.

Continuing the evaluation of the bactericidal effects of *S. mutans*, Nagai et al. [18] formed nine experimental groups, namely: 1. 650 nm laser irradiation; 2. 940 nm laser irradiation; 3. The application of MB; 4. The application of neocyanine blue; 5. 650 nm laser irradiation with MB; 6. 650 nm laser irradiation with Azc; 7. 940 nm laser irradiation with MB; 8. 940 nm with Azc; 9. The untreated control group. The bactericidal efficacy of each treatment examined in this study was different. The combination of PDT with MB and a 650 or 940 nm laser, as well as the combination of Azc with a 940 nm laser and MB, significantly reduced the number of *S. mutans* in infected dentin plaques [18].

Twenty children between 6 and 8 years old with active caries and dentin cavitation, located on the occlusal surface of homologous deciduous molars, showed that a PDT can be used as an additional treatment against cariogenic microorganisms after selective caries removal without compromising composite resin restorations. PDT was performed on selected teeth (n = 20) after selective caries removal, using an InGaAlP laser (TF Premier mm Optics, São Carlos, São Paulo, Brazil) with the following specifications: wavelength 660 nm, light spectrum region red, power 100 mW, energy density 640 J/cm^2^, for 180 s, associated with a 0.005% methylene blue photosensitizer. The pre-irradiation time was 5 min. Then, the teeth were rinsed abundantly with water for 1 min. After treatment, there was a significant reduction in the number of microorganisms after selective caries removal (*p* = 0.04) and after the application of PDT (*p* = 0.01). The reduction in the CFU of *S. mutans* was 76.4% after caries removal, but when associated with PDT, it was 92.6%. After 6 months of clinical evaluation, no differences between the groups were found for retention, marginal adaptation, color, marginal discoloration, and secondary caries [59].

Aiming to investigate the amount of bacterial destruction by riboflavin-mediated PDT around fixed orthodontic appliances using the two bacterial strains *Streptococcus mutans* and *Streptococcus sanguinis*, a total of 80 metallic brackets were divided into four groups consisting of twenty brackets each. Group I: riboflavin + LED irradiation; Group II: riboflavin alone; Group III: immersion in 0.2% chlorhexidine gluconate solution; and Group IV: not subjected to any treatment. All metallic brackets were immersed in the standard bacterial solutions and incubated for 48 h. All samples were subjected to an MTT assay for microbial cell viability testing after treatment. After 24 h of incubation, biofilms that adhered to the mesh of metallic brackets after treatment were assessed using confocal laser microscopy. This laboratory investigation revealed that riboflavin-mediated PDT significantly reduced the amount of *S. mutans* and *S. sanguinis* around the orthodontic brackets [60].

In this in vitro study, the researchers carried out the bacterial inoculation of *E. faecalis* in a 1% Pioktanin blue solution; in the control group, distilled water was added to the microtube. For laser irradiation, a diode laser was used (OPELASER Filio; Yoshida TradeDental Distribution Co., Ltd., Tokyo, Japan) at a wavelength of 808 nm, as well as a flexible fiber delivery system (Φ = 0, 2 mm). Laser irradiation was performed with an output power of 3 W with continuous waves at 6 mm from the bottom of the 1.5 mL microtube for 10, 20, 40, 50, and 60 s. Controls were performed without laser irradiation and with laser irradiation for 60 s without Pioktanin. After irradiation, bacterial counts were performed, and greater reductions in the number of *E. faecalis* colonies were observed with a longer laser irradiation time compared to the control (without laser and Pioktanin). The greatest reductions were seen in the non-laser Pioktanin group compared with laser irradiation for the 10 and 20 s group. Diode laser irradiation in combination with PB as a PS is effective for killing *E. faecalis* without inducing toxicity to HDFa cells. This treatment can be useful for root canal irrigants [17].

*Lactobacillus* acidophilus bacteria present in infected dentin were evaluated in this study to measure the bactericidal effects of the PDT technique with two PS: acid red (AR) and brilliant blue (BB) associated with two wavelengths of light: red and infrared. The groups were divided into two laser irradiation groups (650 nm and 940 nm), two PS groups (BB and AR), and four PDT groups (650 nm laser irradiation with BB; 650 nm laser irradiation with AR; 940 nm laser irradiation with BB; and 940 nm laser irradiation with AR and a control). The irradiation mode was set to continuous wave, the irradiation distance was fixed at 10 mm using a flexible arm, the irradiation time was 60 s, the light delivery was the actual measurement value when using the chip, the 940 laser was 0.6 W, and the 650 laser was 0.009 W. The PDT with the 650 nm laser combined with BB solution was most effective in sterilizing dentin plaques infected with *L. acidophilus* [14].

Fekrazad et al. [61] conducted a clinical trial to evaluate the effects of PDT in reducing *Streptococcus mutans* in children with severe early childhood caries. Twenty-two children aged 3–6 years were selected. TBO powder dissolved in sterile distilled water was used to obtain a final concentration of 0.1%, which was then kept in dark glass. The patients were treated with 10 mL of TBO (0.1 mg/mL) for 1 min and were irradiated by a light-emitting diode (LED), which emitted light at 2000 mW/cm^2^, with a predominant wavelength of 630 nm per 150 s after 10 min considered the pre-irradiation time. Exposure sites involved the buccal, lingual, and palatal surfaces of all teeth and the dorsal surface of the tongue, which was exposed for 30 s using the blunt head of the LED unit. The total energy density was 300 J/cm^2^. After treatment, the *S. mutans* count in saliva decreased significantly after 1 h. However, the difference in the reduction in the *S. mutans* counts in saliva was not significant between the baseline and 7 days after treatment.

Oliveira et al. [62] reported a case in which they evaluated the effect of PDT on the viability of specific groups of microorganisms from the biofilms of dental microcosms on the occlusal surfaces of erupted first permanent molars. Biofilms were irradiated once with visible red-light wavelength (625 ± 30 nm) and a power density of 40 mW/cm^2^, using fluences of 18.75 J/cm^2^, 37.5 J/cm^2^, or 75 J/cm^2^, which correspond to 1.88 J, 3.75 J, and 7.50 J of total light energy. These irradiation parameters were achieved by varying the time of exposure to light (468 s, 935 s, and 1870 s). Then, the biofilms were immediately washed in medium and kept in the dark until sonication. In this study, PDT promoted a significant reduction in microorganisms, with a trend towards a dose-dependent effect.

Carvalho et al. [63] presented a case report of a 9-year-old female patient with deep caries in the lower right first molar who was treated with PDT to neutralize the remaining bacteria. Papacarie Duo^®^ (PD) and PDT were combined. After the application of Papacarie Duo^®^, the carious tissue was carefully removed as the cavity was stained for 1 min with rose Bengal solution, irradiated with high-intensity LED constituting the PDT technique, and definitively restored with composite resin. At the 6-month follow-up, there were no signs of caries, showing the success of the applied techniques. Although it achieved excellent clinical and bacterial reduction results, the approach took an excessive amount of time to manage the carious lesion.

With the aim of evaluating the use of PDT as an adjuvant in the minimally invasive treatment (partial removal) of primary carious tissue, Ornellas et al. [64] recruited 18 children aged 4 to 5 years with primary molars with active lesions of deep caries. The treatment was performed with 100 μg mL of MB solution for 5 min, and then irradiation took place with a low-power laser emitting red light (InGaAIP-gallium–indium aluminum phosphide; with a wavelength of 660 nm; 100 mW; 300 J/cm^2^; and 9 J of energy). After treatment, there was a microbial reduction that varied from 69.88% to 86.29% and was significantly observed for total microorganisms, *Streptococci Mutans*, *Streptococcus* spp., and *Lactobacillus* spp. The authors concluded that PDT presents a promising future for clinical use as an adjuvant for the reduction of microorganisms in all types of dentins.

A randomized clinical trial included 108 homologous permanent mandibular first molars [37,45] with biofilm from 54 children aged 6 to 12 years. PDT was performed (0.01% PS MB/therapeutic laser-InGaAIP) according to the following protocols: group 1: the collection of biofilm from the distal area of the lingual surface 36 µm before PDT; group 2: the mesial area of the lingual surface of 36 µm 1 min after PDT; group 3: the lingual surface area of 46 µm before PDT; and group 4: the mesial area of the lingual surface of 46 µm 5 min after PDT. The authors concluded that pre-irradiation reduced the number of colony-forming units of mature bacterial biofilms in vivo. A time of 5 min resulted in a greater reduction in the number of colony-forming units [65].

Faria et al. [66] carried out a clinical study to evaluate the clinical performance of composite resin restorations in deciduous molars submitted to selective caries removal associated with PDT. The primary molars of patients aged 6 to 15 years with deep carious lesions, without signs and symptoms of pulpal involvement, were included in the study. After treatment, the authors concluded that the marginal adaptation of resin restorations in deciduous molars was positively affected by PDT after 12 months of follow-up.

Nassaj et al. [67] evaluated the effect of PDT with a different PS on the microleakage of composite resin restorations. Seventy-two deciduous teeth with sound labial/buccal and lingual surfaces were collected for this study. The teeth were randomly divided into four control groups: PDT with indocyanine green, PDT with MB, PDT with TBO, and the control group. No significant difference was observed in the microleakage between the groups on the occlusal wall. However, there was a significant difference in the cervical wall between the control and TBO groups and the control and MB groups. There was a significant difference in the microleakage between the occlusal and cervical walls within each group. The authors conclude that PDT can be used in cavities with enamel margins to decrease the microbial load and prevent secondary caries, but PDT is not recommended for cavities with cementum margins. Alternatively, it can be performed with indocyanine green as a photosensitizer in these cases.

### 2.2. Hypoplastic Teeth

With the aim of investigating the microshear bond strength of resin-modified glass ionomer cement bonded to hypoplastic teeth after the application of chlorhexidine (CHX), sodium hypochlorite (NaOCl), Er, Cr:YSGG and PDT mediated by MB, Alshami et al. [68] collected a total sample of 60 erupted and extracted hypoplastic teeth collected from children younger than 16 years who were submitted to different conditioning protocols, including a control group that involved the bonding of hypoplastic teeth. The group that included hypoplastic enamel was treated with 0.2% CHX solution and 2% NaOCl solution for 30 s, followed by rinsing and drying for 5 s; there was also the MB-PDT group, which involved the use of a methylene blue PS, and the Er,Cr:YSGG group. This study concluded that APDT might be a potential therapeutic strategy to increase the microshear bond strength of RMGIC to hypoplastic enamel.

### 2.3. Extrinsic Tooth Staining

Pessoa et al. [69] presented a compelling case study involving a 10-year-old boy who presented with recurrent generalized black spots affecting both permanent and primary teeth, accompanied by gingival bleeding. The treatment approach involved the use of 1% Photodynamic Therapy (PDT) using Toluidine Blue O (TBO) (10 mg/mL) along with 300.18% methylparaben and 30 mL of purified water. These substances were carefully applied to the tooth surfaces using a micro brush. For the irradiation process, an indium gallium aluminum phosphor (InGaAlP) diode laser with a wavelength of 660 nm was employed continuously, delivering 100 mW with a focal point of 600 µm. The treatment consisted of five sessions conducted once a week.

Following the application of PDT, irradiation was administered at 70 J/cm^2^ in non-contact mode for 23 s near the dental surface. The results were highly successful, with complete removal of the black-pigmented spots from the teeth. Notably, there were no reports of spot recurrence during the 7-month follow-up period. Additionally, the study investigated bacterial species (*Actinomycetemcomitans*, *P. gingivalis*, *P. intermedia*, *P. melaninogenica*, and *P. nigrescens*) and found a significant reduction (22%) in the overall prevalence of bacteria post-treatment, particularly for *P. intermedia*, which was not detected in any of the samples [69,70]

### 2.4. Endodontic Treatments

Based on previous studies, Okamoto et al. [71] performed a comparative study of endodontic treatments in deciduous teeth for two groups that were randomly allocated: group I involved patients undergoing conventional endodontic therapy (n = 15), and group II included patients submitted to conventional endodontic therapy combined with PDT (n = 15). The chosen photosensitizing agent was MB (concentration of 0.005%), which was applied inside the canal with a sterile paper cone for 3 min, followed by irradiation of laser light for 40 s (wavelength: 660 nm, density energy: 4 J/cm^2^, power: 100 mW), delivered in direct contact at the root canal entrance. Clinical follow-up investigating fistulas and mobility was performed at 1 and 3 months after endodontic treatment. The reduction in the bacterial load was 93% in group I and 99% in group II, with no statistically significant difference. Conventional treatment combined with antimicrobial PDT with the parameters used in this study proved to be effective, but it showed an equal efficacy to conventional endodontic treatments alone.

Okamoto et al. [66] performed a series of cases with the aim of testing the combination of conventional endodontic therapy in primary teeth with PDT. Five deciduous anterior teeth were evaluated in healthy children aged 3 to 6 years, regardless of their race or ethnicity, with a diagnosis of pulpal necrosis due to caries, in conditions to be restored, and with at least 2/3 of the root remaining. The 0.005% MB solution was inserted into the root canal for three minutes. After three minutes, the paper tips were removed, and the laser was applied with a radiant exposure of 4 J/cm^2^, an output power of 100 mW, and a low-power laser emitting λ = 660 nm for 40 s, leaving the laser tip active in contact with the canal. In this study, no optical fibers were used. The results obtained in this series of cases showed a bacterial reduction from 37.57% to 100%. The authors concluded that PDT can be considered an easy-to-apply alternative that does not generate microbial resistance to act as a support in the decontamination of root canals.

Another case report described the impact on the quality of life and oral health after endodontic treatment associated with PDT in the traumatized deciduous teeth of a 4-year-old female patient who was assisted in a Dental Trauma Care Program considering a period of 12 months of follow-up. The 4-year-old child had discolored teeth, as he had fallen from his own height. The channel was filled with 0.01% MB dye as a photosensitizer for 5 min. Then, a laser fiber, with a wavelength of 660 nm, 100 mW, and 120 J/cm^2^ 4 J, was introduced into the apical portion of the root canal, moving from apical to cervical to ensure the equal diffusion of light into the lumen of the root canal channel for 90 s. The child was very pleased with the result. The clinical and radiographic findings during the 12-month evaluation period showed the absence of a radiolucent area in the periapical region with new bone formation, and the restorations were perfectly adapted. The association of PDT with a conventional endodontic treatment was effective in this case. It allowed the regression of the sinus tract and the formation of new bone. Furthermore, this case report emphasizes the need for and importance of monitoring cases of dental trauma in pediatric dentistry clinics [65].

Silva et al. [72] investigated the action of PDT in controlling pain after endodontic treatment in asymptomatic teeth with a primary infection in a single visit. Sixty single-rooted teeth with pulp necrosis and periapical lesions were selected and randomly divided into two groups (n = 30) according to the protocol: a control group and a group undergoing PDT. The PDT consisted of 0.005% MB as a photosensitizer, using an AsGaAl diode laser, with a wavelength of 660 nm, 100 mW of power, and 9 J of energy, and optical fibers which were 365 μm in diameter. There was a statistically significant difference in the periods of 8, 12, 24, 48, and 72 h between the control group and the PDT group. It is concluded that PDT had a significant effect on decreasing post-endodontic treatment pain in teeth with pulp necrosis and asymptomatic periapical lesions.

### 2.5. Mucositis

In a randomized clinical trial, Medeiros et al. [73] evaluated the effect of low-level laser therapy combined with photochemotherapy for the treatment of chemotherapy-induced oral mucositis in 18 patients aged 3–16 years undergoing chemotherapy or chemotherapy associated with radiotherapy. The treatments were carried out in a hospital bed. The lesion was pigmented with PS (aqueous solution with 0.005% MB). The PS remained over the lesion for five minutes (pre-irradiation period). Then, the lesion was irradiated with red light (wavelength: 660 nm; power: 100 mW; distance: 1 cm above the lesion; and exposure time: 90 s). At the end of the evaluation period, the group that underwent photochemotherapy combined with a low-level laser showed smaller lesion areas, demonstrating a greater therapeutic effect compared to the low-level laser alone. Therefore, photochemotherapy combined with a low-level laser showed a greater therapeutic effect on reducing the severity of oral mucositis compared to the use of laser therapy in isolation.

### 2.6. Diabetes

Mannakandath et al. [74] conducted a clinical trial involving 40 adolescents aged 14 to 19 years with clinically diagnosed type II diabetes. Group I (control) included patients who underwent a single visit of full-mouth ultrasonic desquamation and were re-advised to perform rigorous and regular brushing using a mouthwash with chlorhexidine gluconate (Clorasept, Ri-yadh, Saudi Arabia), and group II (test) was composed of patients who underwent full-mouth US along with three sessions of PDT mediated with adjuvant methylene blue. Group II underwent three sessions of PDT on day 1, day 7, and day 10 on at least three teeth per patient. The three teeth of the participants who suffered from the most severe gingival inflammation were selected and investigated throughout the study, as shown in Figure 1. The photosensitizer methylene blue (MB) at a concentration of 0.005% was used in the present study. The MB photosensitizer was transferred to the gingival sulcus by placing a blunt needle at a 2 mm depth for 10 s. A diode laser (HELBO^®^ TheraLite—Bredent Medical, Senden, Germany) was used with a spot area of 0.028 cm^2^, an exposure time per point of 60 s, energy of 6 J per point, and an output power of 100 mW in continuous wave mode. The laser power, density, and wavelength used were 150 mW, 1.1 W/cm^2^, and 670 nm, respectively. In addition, a flexible (fiber-optic) tip of 0-degree orientation was used to irradiate the buccal and lingual surfaces within the gingival sulcus. There was a statistically significant reduction in the plaque index (Pi) and bleeding on probing (BOP) in both group I and group II from the baseline to 12 weeks of follow-up. There was also a statistically significant difference in BOP when group I was compared with group II at the 12-week follow-up assessment (*p* < 0.05). The HbA1c assessment did not indicate a statistically significant difference within or between the groups at any time point (*p* > 0.05). Both MMP-8 and MIP-1α reported a significant decrease for both groups I and II at 6 weeks and 12 weeks of follow-up compared to the baseline (*p* < 0.05). A comparison between the groups indicated that a statistically significant difference could be observed at the 6-week follow-up that was still maintained at the 12-week follow-up (*p* < 0.05). The logistic regression analysis revealed that even after controlling for mean BMI as a predictor, the change in the biomarker levels, along with the improvement in the plaque scores and bleeding on probing, was not significant (*p* > 0.05). The authors concluded that PDT significantly improved bleeding on probing and pro-inflammatory biomarkers in diabetic adolescent patients undergoing fixed orthodontic therapy.

## 3. Advantages and Disadvantages of PDT

The advantages of using PDT include the elimination of bacteria in a short period of time, reduced incidence of injury to adjacent tissues, access to areas with complex anatomy, low risk of bacteremia in immunocompromised patients and high reproducibility [67,68], with PDT being an alternative antibacterial therapy for plaque-related diseases such as dental caries in children [61].

The atraumatic nature of PDT has implications, especially with regard to the treatment of pediatric and special patients [75]. However, in most cases, its use is still indicated as an adjuvant therapy, thus reducing the number of microorganisms in remaining tissues, and due to PDT and the great clinical and commercial appeal of the technique, two factors hinder the dissemination of the treatment: the cost of medicines and equipment, in addition to the lack of knowledge of the technique by the medical and dental professions. The drugs used for PDT, whose active principles are synthetic, are economically inaccessible to the majority of the population due to difficulties in production and high investment in their development [76]. In view of the literature presented, we can observe that, in fact, the consecration of laser as a therapy requires knowledge of the applied energy, an investigation of the effects it produces in the body and the application of a correct methodology [77,78].

## 4. Conclusions

In conclusion, Photodynamic Therapy (PDT) shows promise as an effective tool against bacteria, fungi, and viruses in pediatric dentistry. With user-friendly protocols tailored for each application, PDT seamlessly integrates into clinical practice, supported by ongoing research to optimize its implementation. In pediatric dentistry, PDT can be an effective option for treating various oral conditions in children. Some of the benefits of Photodynamic Therapy in pediatric dentistry have been outlined in this review and include minimal invasiveness, bacteria reduction, caries treatment, endodontic treatment, and mucositis, among others. Adopting this innovative approach has the potential to transform dental practice, enhancing oral health and patient care for a better future. Further in-depth studies on dosimetric parameters related to light sources and photosensitizers are still needed to determine optimal protocol choices.

## Data Availability

Not available.
